# Detection of Audio Tampering Based on Electric Network Frequency Signal

**DOI:** 10.3390/s23167029

**Published:** 2023-08-08

**Authors:** Hsiang-Ping Hsu, Zhong-Ren Jiang, Lo-Ya Li, Tsai-Chuan Tsai, Chao-Hsiang Hung, Sheng-Chain Chang, Syu-Siang Wang, Shih-Hau Fang

**Affiliations:** 1 Forensic Science Division, Ministry of Justice Investigation Bureau, New Taipei City 231, Taiwan; m38025@mjib.gov.tw; 2 Department of Electrical Engineering, Yuan Ze University, Taoyuan 320, Taiwan; s1080543@mail.yzu.edu.tw (Z.-R.J.); s1080516@mail.yzu.edu.tw (L.-Y.L.); s1080518@mail.yzu.edu.tw (T.-C.T.); s1108501@mail.yzu.edu.tw (C.-H.H.); s1104633@mail.yzu.edu.tw (S.-C.C.)

**Keywords:** detecting tampered audio, ENF signal, wavelet decomposition, AR model, Chinese ENF database

## Abstract

The detection of audio tampering plays a crucial role in ensuring the authenticity and integrity of multimedia files. This paper presents a novel approach to identifying tampered audio files by leveraging the unique Electric Network Frequency (ENF) signal, which is inherent to the power grid and serves as a reliable indicator of authenticity. The study begins by establishing a comprehensive Chinese ENF database containing diverse ENF signals extracted from audio files. The proposed methodology involves extracting the ENF signal, applying wavelet decomposition, and utilizing the autoregressive model to train effective classification models. Subsequently, the framework is employed to detect audio tampering and assess the influence of various environmental conditions and recording devices on the ENF signal. Experimental evaluations conducted on our Chinese ENF database demonstrate the efficacy of the proposed method, achieving impressive accuracy rates ranging from 91% to 93%. The results emphasize the significance of ENF-based approaches in enhancing audio file forensics and reaffirm the necessity of adopting reliable tamper detection techniques in multimedia authentication.

## 1. Introduction

In recent years, audio files have become increasingly prevalent in daily life. However, these sound files may be tampered with and distributed via the Internet or submitted to the court as evidence after being maliciously edited. Such scenarios can lead to serious social issues, highlighting the indispensable role of audio file authenticity recognition in the judicial system [1]. Typically, edited audio files are difficult to detect by the human ear; therefore, this project aims to implement a method for detecting whether an audio file has been tampered with.

This project aims to detect the authenticity of audio files by analyzing the Electric Network Frequency (ENF) signal. In most countries, the ENF—which refers to the frequency of the alternating current supply from power generators, transmission, and distribution systems, to household electrical appliances—is fixed at 60 Hz. In areas with alternating currents, the ENF signal can be captured using recording devices. The uniqueness of the ENF signal in the audio file, and its abnormal variation due to editing or tampering, makes it a reliable indicator of the authenticity of the audio file [2,3,4,5,6]. We conducted a thorough investigation of Electrical Network Frequency (ENF)-based applications in media forensics. To support our research, we referenced some relevant papers. The first paper presents a comprehensive review of ENF-based applications in various aspects of media forensics [7]. The second paper proposes a Conv-Attention Network for detecting the presence of ENF signals in short-duration audio, offering insights into signal detection techniques [8]. The third paper, investigates the robustness of ENF signals as a fingerprint for digital media authentication, providing valuable insights into the reliability of ENF-based authentication methods [9]. By drawing upon these reputable sources, our study benefits from the latest advancements and knowledge in the field of ENF-based applications in media forensics.

In the past, there have been studies using ENF signals to identify the authenticity of audio files. It was demonstrated that the ENF signal is an effective tool for verifying the authenticity of audio signals [3]. Subsequently, improvements were made based on the least square error [5] and using an AR model [6]. A method was proposed in [10] to detect the authenticity of audio files based on the discontinuity of the ENF phase, which was further improved in [11]. ENF variations were compared with predefined thresholds and evaluated using unsupervised learning [12]. Then, the detection criteria were subsequently modified for improved performance [13]. However, the accuracy of past methods was still affected by noise and compression rates [14,15]. A new method in [16] showing outperformed the previous.

The difference between our method and [16] is that we used additional learning models in the final step. Moreover, the dataset and language in this paper are totally different to those in the literature. In the experiment which uses our Chinese ENF database’s results, the accuracies of the three classification models were similar, indicating that KNN/DNN are also suitable for this method. In addition, before constructing the database, we investigated the impact of different audio factors on ENF extraction and the accuracies of the models. We observed that the length of the audio file does not have a direct relationship with the accuracy, indicating that the audio file duration suitable for this method is not limited to 19–39 s as described in [16]. However, in investigating the relationship between the method of audio file tampering and accuracy, the experiment of different sentence splicing tampering had lower accuracy than that of deleting or copying the same sentence, which can be improved in the future.

## 2. Research Process

**Figure 1:**
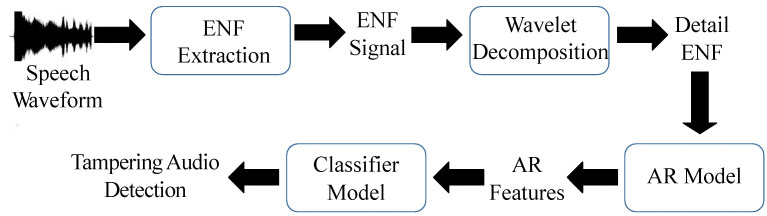
Experimental procedure diagram.

The experimental procedure of this project is shown in Figure 1. Firstly, we analyzed the influence of different environments and devices on ENF extraction from audio. Then, we recorded a speech database and simulate several common audio tampering methods. After the Chinese database was constructed, we apply ENF extraction from audio and wavelet decomposition for detailed ENF changes. Then, the AR coefficients were generated by the AR model, and finally, the SVM/KNN/DNN classification models were trained using the AR feature coefficients to distinguish the authenticity of audio files.

### 2.1. Related Work

Before experimenting, we searched for information on ENF signals and compiled the following characteristics.

ENF (Electric Network Frequency) is the frequency of the AC power supply in the power grid. All generators, transmission and distribution equipment, and users in the same power grid use this frequency. Most countries have a fixed power grid frequency, such as 50 Hz in Europe (e.g., the UK, France) and 60 Hz in the US, most other American countries, and Taiwan. In regions with AC power, ENF signals can be captured by recording devices, and because of their uniqueness in the power grid, the frequency changes in multimedia files can be analyzed to identify whether the file has been tampered with [3,5,6].

The power supply of recording devices can be divided into two types: direct power supply (such as desktop computers and landline phones that directly use household AC power) and battery power supply (such as laptops and mobile phones). It is generally believed that the recorded ENF is related to the internal circuit design and electromagnetic compatibility of the recording device, which is indeed the case for devices with direct power supply [17]. However, the ENF signal captured by battery-powered recording devices is unrelated to the electromagnetic field. Even if not connected to a power source, if the device is close enough to other AC appliances (such as electric fans and refrigerators), it may capture the ENF signal generated by the AC appliances [17,18]. Moreover, under the same environment, the strength of the captured ENF may vary depending on the device used [18].

In addition to 50 or 60 Hz, multiples of 50 or 60 Hz may also have ENF signals [11,18]. In a sound file, for example, traces of ENF signals may appear at 60, 120, 180, 240, and so on. Some recording devices may not be able to capture low-frequency signals (such as 60 Hz ENF) due to design limitations [19], but the harmonic signals of their multiples can also be analyzed.

### 2.2. ENF Extraction

Since the discovery that 50 or 60 Hz electric network frequency (ENF) signals can be captured from recordings [2], numerous studies have used this discovery to evaluate the authenticity of audio files. ENF is a stable and unique signal in the electric network, and a method [3] was proposed to verify the authenticity of audio files by detecting the discontinuity of ENF. This method has since progressed, and it was found that harmonic signals at multiples of 50 or 60 Hz can also be used to distinguish between authentic and manipulated audio files [11]. Since the ENF signal in Taiwan fluctuates at around 60 Hz, the audio file is sliced into one-second frames with a sampling rate of 500 Hz and overlap rate of 0.9, and the desired frequency band (i.e., 60 Hz) is extracted.

In FFT, the audio signal is first sampled and N samples are collected into one observation unit called a frame. To avoid drastic changes between adjacent frames, there is an overlap region containing M samples, where M is the product of the number of FFT points N and the overlap rate. Here, the sampling frequency fs is set to 500 Hz, each frame is one second long with an overlap rate of 0.9, and the number of FFT points N is 4096 [16], meaning the original audio signal is zero-padded to have a fixed number of 4096 points in each frame.

If the measured signal is a periodic signal and there is an integer number of periods within the sampling time, the waveform at the two ends in time is continuous. However, if it is for non-periodic signals, FFT will produce some energy distribution that does not exist in the original signal in order to conform to the discontinuous changes at the left and right ends. The boundary of the finite sequence sampled will show discontinuity, causing errors in analysis. Windowing can be used to eliminate the amplitude of the left and right ends of the audio frame as much as possible and reduce the errors generated by performing FFT on non-integer periods. In this case, we use the Hamming window function, which can be represented by the following Equation (1).

(1) $$w\left(n\right)=a_{0}-\left(1-a_{0}\right)\cos(\frac{2\pi n}{N-1})\;,\;0\leq n\leq N-1$$

where w(n) represents the value of the Hamming window function at index *n*, *N* is the total number of data points in the window, and $a_{0}$ is a parameter that determines the shape of the window. The Hamming window is commonly used in signal processing and serves to reduce spectral leakage and improve the frequency resolution of the analyzed signal. The window smoothly tapers the data towards the edges, reducing artifacts that might occur during the processing of finite-length data sequences.

Different values of $a_{0}$ will have different effects. Taking $a_{0}=0.53836$ is known as the Hamming window, and the Hamming window can be simplified as shown in Equation (2), where the audio frame length *L* is N+1.

(2) $$w(n)=0.54-0.46\;\cos(\frac{2\pi n}{N})\;,\;0\leq n\leq N$$

An ideal bandpass filter only allows signals within a certain frequency band to pass through. In this case, we only want to keep the signals within the 60 ± 0.5 (59.5–60.5) Hz frequency band.

Our purpose of ENF extraction is to capture abnormal ENF variations rather than precise ENF details. Therefore, we adopt a weighted averaging calculation method proposed in [20]. As depicted in Equation (3), the process begins with the extraction of the frequency band with L1 = 59.5 Hz and L2 = 60.5 Hz from the frequency domain signal. Subsequently, a weighted average calculation is performed on the signal. For each frequency *f* in every frame of the signal, the average value is computed by applying a weighting factor based on the energy *S*. Here, f(n,l) denotes the frequency of the lth point in the signal within the nth frame, and it is multiplied by the energy |S(n,l)| corresponding to that specific point, acting as the weight. Each point in the signal undergoes a separate calculation, where they are multiplied by their corresponding weights and then summed together. Finally, the sum of the weighted values is divided by the sum of the weights to yield the weighted average value of the signal frequency *f*. For instance, if the duration of each frame is 1 s, and the frame overlap rate is 0.9, 9 ENF signal values can be computed for 1 s of audio.

(3) $$x(n)=\frac{\sum_{l=L1}^{L2}f\left(n,l\right)\left|S(n,l)\right|}{\sum_{l=L1}^{L2}\left|S(n,l)\right|}$$

### 2.3. Wavelet Decomposition

Wavelet decomposition [21] is used to remove noise and generate a detailed ENF signal. The concept is to analyze the input signal’s detail using iterative wavelet transforms. The result of each level of decomposition is that the low-frequency signal obtained from the previous level is further decomposed into low-frequency and high-frequency components.

### 2.4. AR (Auto Regressive Model) Model

As shown in Equation (4), the Auto Regressive model [6,22] predicts future behavior based on past behavior [23]. The equation expresses the computation of the detail ENF signal at time index *n* as the sum of the product of AR coefficients $a_{i}$ with their respective detail ENF signals d(n−i), for *i* ranging from 1 to *m*. The result is then combined with the prediction error e(n).

- d(n): the *n*th detail ENF signal.
- $a_{i}$: the *i*th AR coefficient.
- *i*: from layer 1 to layer *m* (*m* is set to 14 [16]).
- e(n): prediction error.

(4) $$d\left(n\right)=\sum_{i=1}^{m}a_{i}d\left(n-i\right)+e\left(n\right)$$

### 2.5. Training Different Classification Models

After generating the AR coefficients, the next step is to train the models. This project uses three different models for classification training: Support Vector Machine (SVM) model, K Nearest Neighbor (KNN) model, and Deep Neural Networks (DNN) model.

SVM (Support Vector Machine) is a supervised learning algorithm based on statistical learning. It finds a hyperplane that separates two different sets. The general classification goal is to find a dividing line between different data categories. In general, this dividing line is very complex and there are many possibilities. However, SVM aims to find the best solution among these many possibilities. The spirit of the SVM algorithm is to find a dividing line so that the points on the boundary are as far apart as possible, making the model more resistant to noise. The linear SVM finds a line in the plane that maximizes the distance between the line and the data points of different categories.

However, in reality, not all data can be separated by linear means. In this case, a kernel function is used to project the data nonlinearly into a higher-dimensional space, making it easier to separate data into different categories. We use RBF (Radial Basis Function, Gaussian transformation) [24] as a nonlinear kernel function.

In classification problems, the KNN (K Nearest Neighbor) [25] algorithm uses the majority voting criterion to determine which class a new data point belongs to by considering the k-nearest neighbors. The user first decides the value of k, then the algorithm calculates the distances between the new data point and all the other data points and identifies the k nearest neighbors. The algorithm then checks which class has the majority among these k neighbors, and assigns the new data point to that class. It is generally recommended to choose an odd value of k to avoid ties between classes.

DNN (Deep Neural Network) is the simplest multi-layer neural network model [26], including an input layer, 7 hidden layers, and an output layer. All layers are fully connected, and by adding more hidden layers, the learning ability of the model can be improved. Due to the relatively simple structure of the model, a large amount of training data is required to achieve better results. In a neural network, the only operations between neurons are the multiplication and addition of weights, which means that the input and output are linearly related in nature. Therefore, we use the commonly used activation function ReLU as the hidden layer to introduce nonlinearity and solve nonlinear problems. Finally, we use Sigmoid as the output layer. In addition, InitialLearnRate is set to 0.0001, MaxEpochs equaled to 100, and MiniBatchSize is 10 to be our parameters.

As shown in Equation (5), accuracy is mainly used in classification problems to obtain the appropriate percentage of the models being executed to know how much of them are correctly predicted. In any model, there will be several classification problems present when we execute them, so accuracy plays the main role when talking about whether all the problems are correctly predicted or not with respect to the total number of predictions made by the model. We can calculate the accuracy of any model by dividing the correctly predicted problems by the total number of predictions made.

(5) $$\mathrm{Accuracy}=100\%\times \frac{\mathrm{TN}+\mathrm{TP}}{\mathrm{All}\mathrm{testing}\mathrm{samples}}$$

To accurately test this, we use the cross-validation method by dividing the data into multiple subsets and alternately using one part as the test set and the rest as the training set. This allows for a more comprehensive evaluation of the model’s performance. Additionally, we ensure the randomness of the test data selection in each trial to ensure the accuracy of the model.

## 3. Experimental Setup and Database

This chapter introduces the speech databases, our environmental sound database, and the recording and tampering methods for these databases.

### 3.1. Chinese ENF Databases

Before constructing the Chinese ENF database, we referred to the information from the Carioca 1 database [16] as a reference. Carioca 1 is a Portuguese telephone recording database in an office environment with a sampling rate of 44,100 Hz and the ENF signal at 60 Hz. It contains 100 original audio files, 50 audio files modified by deletion, and another 50 audio files modified by copying. Each original audio file has a length between 25 and 33 s, and each modified segment is 2 to 3 s long.

**Figure 2:**
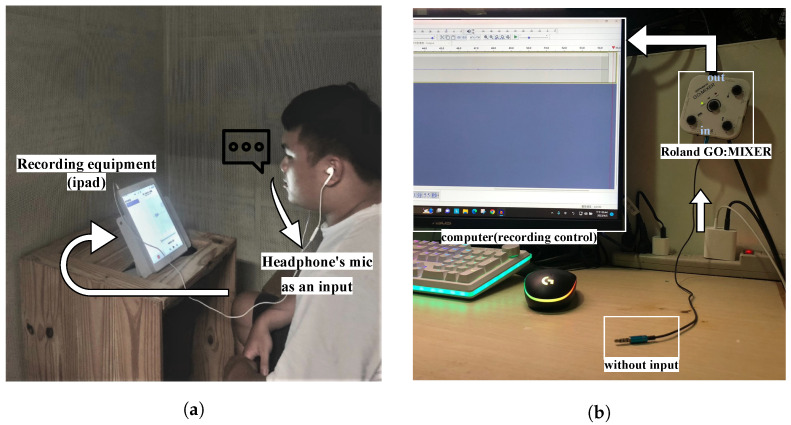
Recorded audio file’s environment. (**a**) Recording ENF signal audio with Chinese voice in an anechoic chamber to be our Chinese ENF database. (**b**) Using GO: Mixer recording ENF signal audio without passing by air in the general environment.

The Chinese ENF database in which we recorded a speech database based on the text of TMHINT (Taiwan Mandarin Hearing In Noise Test) consisting of 320 sentences in an anechoic chamber with and without the recording device, an iPad with an external in-ear microphone, connected to the power socket. To minimize ENF signal interference from other electrical devices in the environment, we turned off the lights in the anechoic chamber during the recording sessions, as shown in Figure 2a. The audio was sampled at a frequency of 44,100 Hz with the ENF signal at 60 Hz band. To standardize the duration of the recordings, we segmented the original audio files into 5 s segments, resulting in a total of 1920 segments. We separated the data for the three tampering types, with the first two types of deletion and copying using the same as those in [16], including 1920 original and modified audio files for each type. The third type is the different audio exchange and includes 1650 original and modified audio files. Each original audio file has a length of 5 s, and each modified segment is 1 s long.

We created tampered audio files in our Chinese ENF database, using three types. Same-signal tampering includes deletion and copy. Deletion involves removing a segment from the middle of an original audio file and merging the remaining two parts to create a tampered audio file with a splice point slightly shorter than the original. Copy involves copying a segment from the middle of an original audio file and pasting it to the end, creating a tampered audio file with a splice point slightly longer than the original. Different-signal is a tampering based on different power supplies. For exchange based on different recording devices, we exchanged the final segments of two audio files of the same length and recorded them with the same device but with different power supplies, creating two tampered audio files with splice points and lengths identical to the original audio files. This summarizes all the data we used and how to create our setup in our Chinese ENF database.

In addition, to ensure the correctness of the experimental process from ENF extraction, wavelet decomposition, and AR model to SVM classification model, we first followed the method of [16] and compared our results with theirs. Ref. [16] used the Carioca 1 and Spanish database, randomly selecting 40% as the training set and the remaining 60% as the test set, achieving an accuracy rate of 97.5% in distinguishing the authenticity of audio files. Our database was constructed through random selection and utilized 5-fold cross-validation. By employing the same training/testing ratio of models as in [16], we achieved an accuracy of 90%, demonstrating the correctness and feasibility of our approach.

### 3.2. Acoustic Environment

Before constructing the Chinese ENF database, we first recorded environmental sounds to investigate the existence of ENF in audio and the effect of different factors on ENF extraction in Section 3.3–Section 3.5. Furthermore, we recorded audio in both connected and disconnected socket states to confirm the presence of ENF signals in the audio. During each recording session, we turned off surrounding appliances such as air conditioners and fans that could produce noise, in order to minimize other ENF signals in the recording. We then analyzed the effects of different environments and devices on ENF extraction, as a reference for selecting the environment and devices for subsequent Chinese ENF database creation.

We recorded ambient sounds in two environments: a typical indoor residential area and an anechoic chamber in our university laboratory. We recorded with devices connected and disconnected to power sockets. In Figure 2b the typical indoor residential area, we recorded 10 min of environmental sound using an iPad external in-ear microphone and 30 min using a laptop with a Roland GO: Mixer which was further connected to an audio source cable. In the anechoic chamber, we recorded 10 min of environmental sound with four devices: the built-in microphones of a Samsung phone, a laptop, and an iPad, as well as an external in-ear microphone connected to an iPad.

### 3.3. Verification of ENF Existence

To verify the existence of the ENF signal, we compared two devices recording 10 min of ambient sound indoors in a general residential area, one connected to a power outlet and the other not. During recording, we turned off surrounding appliances such as air conditioners and fans that could produce noise, in order to minimize other ENF signals in the recording. The two devices compared were an iPad with an external in-ear microphone and a laptop with a Roland GO: Mixer which was further connected to an audio source cable. For the latter device, the laptop was used as the recording device, and the audio source cable was used as the receiver. As the audio input of the GO: Mixer was connected only to the audio source cable, without any other device connected, the audio was not transmitted through the air, aiming to eliminate as much noise as possible.

**Figure 3:**
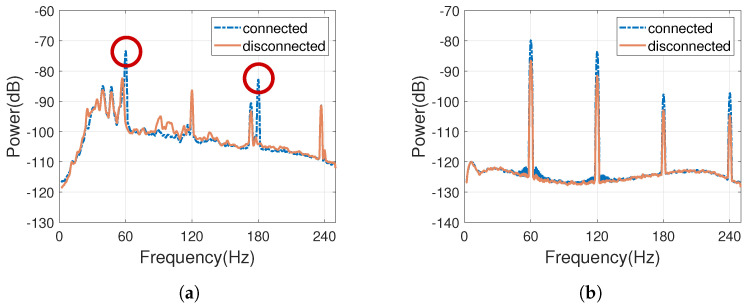
A comparison of the spectrum magnitude of the audio recorded by two devices, with and without being connected to a power socket, in a typical indoor environment in a residential area. The recording (**a**) was from an iPad with an external in-ear microphone, while (**b**) was from a laptop with GO: Mixer further connected to an audio source cable.

Figure 3 illustrates the magnitude spectrum analysis of the recorded audio. In Figure 3a, when the iPad was connected to the power socket and used an external in-ear microphone, it exhibited significantly higher energy levels at 60 Hz and 120 Hz. On the other hand, Figure 3b depicts the spectrum analysis of the laptop with the GO: Mixer, which was further connected to an audio source cable as the recording device. In this case, the laptop demonstrated much lower noise energy compared to Figure 3a. However, when the laptop was connected to the power socket, there was an increase in energy at 60 Hz and its harmonics frequencies, with a noticeable energy accumulation around 60 Hz and 120 Hz. Both experimental results are consistent with the 60 Hz frequency of the power grid in Taiwan. When the device was connected to a power source, there was strong energy at 60 Hz or its harmonic frequencies in the recorded audio, which is the Electromagnetic Network Frequency (ENF) signal. This not only verifies the possible existence of ENF signals in recorded audio but also confirms that ENF signals may exist in frequency bands that are multiples of 60 Hz.

### 3.4. Environment Selection

**Figure 4:**
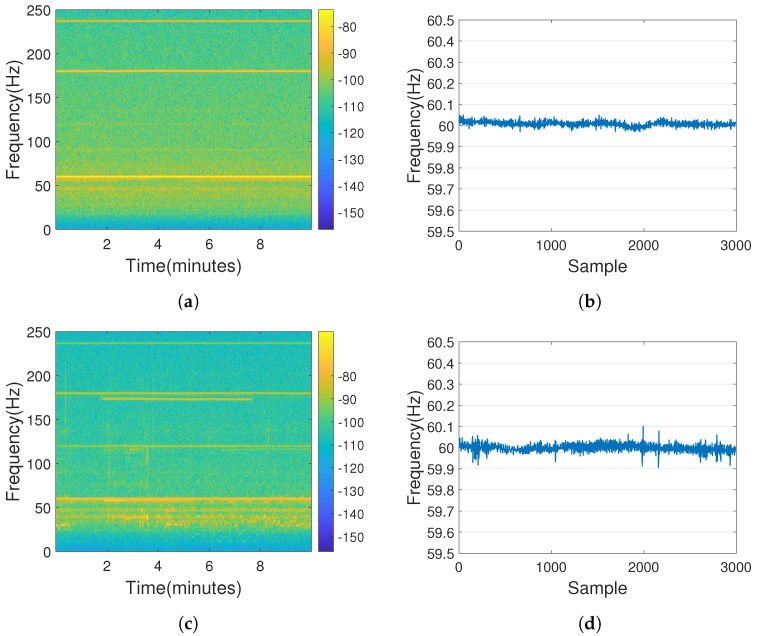
A comparison of ENF extraction from audio recorded separately in an anechoic chamber (**a**,**b**) and a typical indoor environment (**c**,**d**) using the same equipment, which is an iPad with an external in-ear microphone.

As the ENF signals and noise strength recorded in different environments are different, the extraction results of audio recorded with the same equipment in different environments were first compared. Figure 4a,c show the comparison of the audio spectra recorded by an external in-ear microphone of an iPad in two different environments: a typical indoor residential area and an anechoic chamber in our university laboratory, both when connected to the socket. Figure 4b,d display the ENF extraction results of the audio recordings. To eliminate potential noise from other electrical appliances, such as air conditioners and fans, the recording was conducted with those devices turned off as much as possible. Figure 4a,c show that 60 Hz has stronger energy, which is the ENF signal, and the traces of ENF signals can be found in the multiples of 60 Hz. Comparing the two figures, there is more noise in the low-frequency part in the typical indoor environment than in an anechoic chamber environment using the same equipment. In Figure 4b,d, it can be seen that the ENF extraction from the recorded audio in the anechoic chamber environment is more stable than that in the typical indoor environment, so we chose the anechoic chamber as the recording environment for our Chinese ENF database.

### 3.5. Equipment Selection

Since each device has different capabilities in presenting tampering signs, a simple method was used to compare the ENF extraction ability of different devices before establishing the database. The comparison method involved tampering the audio using the same method, with 10 tampering points in the tampered audio. The number of splice points that could be presented by the ENF extraction in recordings from different devices was observed.

**Figure 5:**
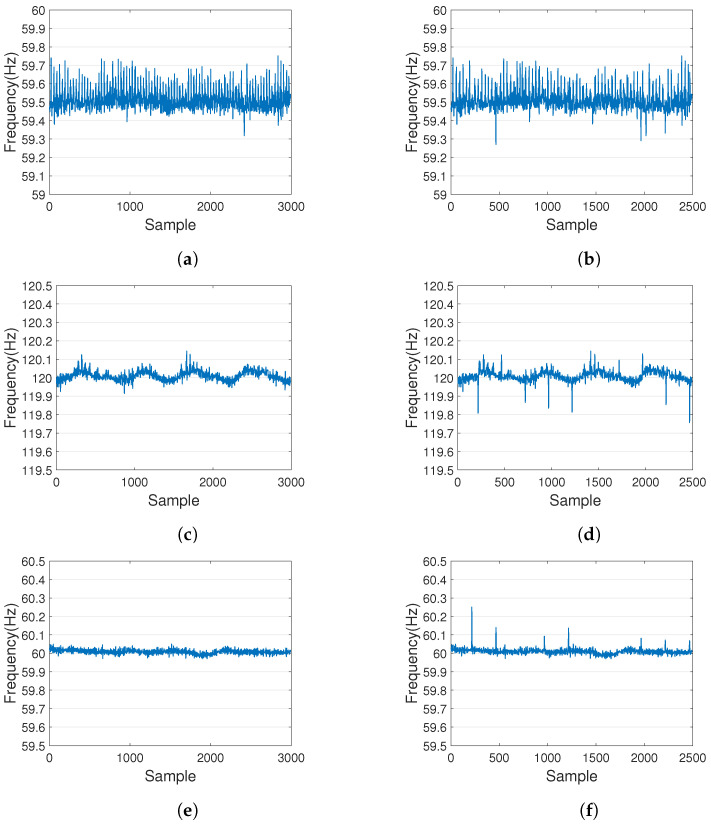
A comparison of ENF extraction from the audio recorded separately by a Samsung mobile with its built-in microphone (**a**,**b**), an iPad with its built-in microphone (**c**,**d**) and the external in-ear microphone (**e**,**f**). (**a**,**c**,**e**) are from the origin audio. (**b**,**d**,**f**) are from the tampered audio.

Figure 5 compares the ENF extraction from the ambient audio recorded by three devices respectively when they were connected to the power socket in an anechoic chamber environment, as well as the ENF extraction from tampered audio. The ENF extraction from the original audio in the left half shows a smoother variation. In the right half, the ENF extraction shows abnormal variations which correspond to the tampered points in the audio.

It can be seen that the effect of extracting ENF from different recording equipment varies greatly. Figure 5a–d compare the ENF signals extracted from recordings made by two different mobile devices, with the iPad recording showing significantly better extraction performance than the Samsung phone. Figure 5c–f compare recordings made by the same recording device with different microphones: the built-in microphone of the iPad and an external in-ear microphone. Although the number of visible junction points extracted was comparable, the ENF extraction from the iPad recording with an external in-ear microphone was more stable than that from the built-in microphone. Therefore, we chose the iPad with an external in-ear microphone as the recording device for building the database.

## 4. Experiment and Result

### 4.1. Experiment 1—Detection of Chinese Tampering Audio Based on Chinese ENF Database’s ENF Signal

This section attempts to use real human speech in Chinese audio signals for authenticity detection. We aim to verify whether our authenticity detection methods are feasible for more realistic recordings.

We recorded our own Chinese voice audio signals using an iPad as the recording device and an external microphone to reduce noise during the recording process. The Chinese ENF database which created ourselves includes recordings from three people to increase the diversity of the experimental data. A schematic diagram of the recording environment as shown in Figure 2a. The iPad is the recording machine that has plugged-in and unplugged different audio files. And the recorder in the anechoic chamber put on an external in-ear microphone, connected to iPad, reads the TMHINT script. Each audio file is 5 s long, there are three methods of tampered audio files (deletion, copy, and different audio tampering), each with one tampered point. According to the experimental procedure, each of the audio files go through ENF extraction, wavelet decomposition, and AR model to obtain AR coefficients. Then we used three different classification models as we just mentioned in the second paragraph to observe the prediction accuracy. During training, 20% of the database was randomly selected as the test set, and the remaining 80% was used as the training set. Both the training and test sets consisted of equal amounts of original and tampered audio files.

In Table 1, we present the results obtained from the Chinese ENF database, which was recorded and extracted by our team. To validate the data, we utilized a five-fold cross-validation method by dividing the data into multiple subsets and alternately using one subset as the test set and the remaining subsets as the training set. This approach enables a more comprehensive evaluation of the model’s performance. Furthermore, we ensured the randomness of the test data selection in each trial to guarantee the model’s accuracy. The table displays the accuracy of three different tampering methods on the Chinese corpus when trained and predicted using the models. Remarkably, all three tampering methods, including our additional method “copy” and various signal tampering techniques, achieved prediction accuracy above 90%. This finding indicates that the method utilized in our Chinese ENF database’s experiment effectively distinguishes between original and tampered audio signals containing Taiwanese human voices with high accuracy.

In Experiment 1, we concluded that using our Chinese ENF database to extract the ENF signal and then decompose it through wavelet to obtain a more robust, detailed ENF signal, entering into the AR model to obtain AR coefficients, and then using machine learning, would improve the accuracy of predicting the authenticity. Our experimental method with our Chinese ENF database can successfully identify the authenticity of Chinese corpus audio files at 91% to 93% accuracy. Sharing the same trend with [16], our own additional KNN model and the DNN model also have similar performances as [16]’s SVM training accuracy. Therefore, extracting audio files using ENF signals makes it possible to distinguish original and tampered audio signals in real-life situations.

### 4.2. Experiment 2—Comparison of Whether Audio Length Affects Experimental Accuracy

In this study, our primary objectives are to detect audio tampering using the Electric Network Frequency (ENF) signal and to investigate the impact of various factors, such as file length and audio tampering methods, on the authenticity detection of ENF signals. To achieve these objectives, we compare our method with the approach presented in [16], where the audio files used in their experiment were limited to a specific range of 19 to 39 s in length. However, we acknowledge that in real-world scenarios, audio files may vary in duration. Therefore, we aim to explore the effects of different audio file lengths on the accuracy of ENF-based authenticity detection.

To address the research gap, we conducted Experiment 1 using our own recorded audio files, following the standard of [16]. We recorded clean acoustic environment audio data using the Roland GO: Mixer instrument and created audio files of various lengths: 5, 15, 25, 35, and 45 s. We then applied three different audio tampering methods and three classification methods to predict authenticity. The experiment’s purpose was to evaluate the accuracy of the results and observe whether audio length influences the detection of ENF signals’ authenticity and whether different tampering methods produce varying outcomes.

The experiment’s accuracy is presented in Table 2. As is evident from the table, the highest accuracy rate is achieved for audio files with a duration of 5 s. This observation leads us to believe that shorter audio files tend to yield better results in identifying tampered audio files.

### 4.3. Expriment 3—Comparison of the Number of Splice Points in a Tampered Audio Files Affects Experimental Accuracy

In addition, we also considered the differences between tampered and original audio files regarding the number of splice points in a segment of audio. In Experiment 1, we used tampered audio files with only one splice point. However, in real-world scenarios, tampered audio files may have different numbers of splice points, which can affect the complexity of the data and influence authenticity prediction accuracy.

To explore this aspect, we conducted an additional experiment using the same recording equipment and three different tampering methods, along with three classification methods to predict authenticity. For each splicing method, tampered audio files with 1, 3, 5, and 7 splice points were created. However, in real life, if there are many splice points in an audio file, it will be intermittent and almost perceptible to the human ear, which is not fair to identify the authenticity of the audio file. Therefore, the final result of this paper is still to use only one splice point.

The results of this experiment are presented in Table 3, and they revealed that tampered audio files with 7 splice points achieved the highest accuracy in predicting authenticity compared to those with fewer splice points. This finding suggests that an audio file with more splice points exhibits a greater difference between the waveform of the tampered audio and the original audio. Consequently, this difference has a significant impact on the AR coefficients used in classification prediction, leading to higher accuracy in predicting authenticity.

However, it is important to consider the practical implications in real-life situations. If an audio file contains many splice points, it may result in an intermittent and almost perceptible signal to the human ear, making it challenging to accurately identify the authenticity of the audio file. Therefore, the final conclusion of this study is to use only one splice point for more reliable and practical authenticity prediction.

## 5. Conclusions and Future Directions

In this research, we successfully established a comprehensive ENF database comprising Chinese voice recordings and evaluated three distinct tampering methods. The results demonstrated that all tested approaches effectively utilized the ENF signal to accurately predict the authenticity of audio signals. This confirms the feasibility of employing the ENF signal for Chinese audio authenticity identification. Moreover, we examined the impact of file length and the number of splice points on recognition accuracy, revealing that shorter files with more splices tend to yield more reliable tampering determinations. The proposed method holds promise for various applications requiring audio authentication, such as judicial judgments and police investigations. However, it is important to acknowledge that the content and complexity of audio signals can influence authenticity identification outcomes, and further research should address these considerations. Furthermore, it is worth noting that the simplicity of the database used in this study compared to real-life general recordings may limit the generalizability of the findings. Hence, there exists ample room for additional research and exploration in the field of acoustic forensics. To advance the field, future investigations could focus on enhancing the robustness of the ENF-based approach, considering diverse and complex audio scenarios. Additionally, incorporating larger and more diverse databases will aid in strengthening the reliability and applicability of audio tampering detection techniques. By addressing these limitations and pursuing new avenues of research, we can fortify the foundation of acoustic forensics, enabling its broader utilization and significance in real-world audio authentication challenges.

## Figures and Tables

**Table 1:** Chinese tampering audio detection accuracy, the data for which is from our Chinese ENF database. Compared with [16], there is the same trend, different countries’ ENF bands, and different languages, and we successfully predicted the authenticity of audio files, achieving accuracies for the three kinds of tampering method’s of above 90%.

	Average Accuracy (%)
Deletion	91.04 ± 2.009
Copy	93.39 ± 2.492
Different signal tampering	92.04 ± 2.059

**Table 2:** Different audio lengths’ accuracy (%). Verifying the impact of different audio file lengths on the accuracy of the prediction, it can be seen that the accuracy rate is highest for a file of 5 s length.

	5 s	15 s	25 s	35 s	45 s
Deletion	99.11	97.78	94.44	84.45	85.56
Copy	96.67	94.44	86.67	81.11	90.00
Different signal tampering	97.78	95.56	93.33	97.78	98.89

**Table 3:** Different splicing points in a tampered audio file’s accuracy (%). Verify the impact of the number of splice points on the accuracy of the prediction, it can be concluded that the more splice points there are, the higher the prediction accuracy is.

	1 Point	3 Points	5 Points	7 Points
Deletion	89.32	94.64	95.81	98.23
Copy	95.82	97.01	98.83	100
Different signal tampering	93.51	94.64	97.03	100

## Data Availability

Due to its proprietary nature <or ethical concerns>, supporting data cannot be made openly available.

## References

[1] Gupta S., Cho S., Kuo C.C.J. (2012). Current Developments and Future Trends in Audio Authentication. IEEE Multimed..

[2] Grigoras C. (2005). Digital audio recording analysis–the electric network frequency criterion. Int. J. Speech Lang. Law.

[3] Brixen E.B. Techniques for the authentication of digital audio recordings. Proceedings of the 122nd Audio Engineering Society Convention.

[4] Maher R.C. (2009). Audio forensic examination. IEEE Signal Process. Mag..

[5] Huijbregtse M., Geradts Z. Using the ENF criterion for determining the time of recording of short digital audio recordings. Proceedings of the Computational Forensics: Third International Workshop.

[6] Garg R., Varna A.L., Wu M. Modeling and analysis of electric network frequency signal for timestamp verification. Proceedings of the IEEE International Workshop on Information Forensics and Security (WIFS).

[7] Güneş A.S., Vatansever S. A Review of Electrical Network Frequency (ENF) Based Applications in Media Forensics. Proceedings of the 2023 5th International Congress on Human-Computer Interaction, Optimization and Robotic Applications (HORA).

[8] Li Y., Lin X., Qiu Y., Zeng H. A Conv-Attention Network for Detecting the Presence of ENF Signal in Short-Duration Audio. Proceedings of the 2022 IEEE 24th International Workshop on Multimedia Signal Processing (MMSP).

[9] Poredi N., Nagothu D., Chen Y., Li X., Aved A., Ardiles-Cruz E., Blasch E. Robustness of Electrical Network Frequency Signals as a Fingerprint for Digital Media Authentication. Proceedings of the 2022 IEEE 24th International Workshop on Multimedia Signal Processing (MMSP).

[10] Nicolalde Rodriguez D.P., Apolinario J.A., Biscainho L.W.P. (2010). Audio Authenticity: Detecting ENF Discontinuity With High Precision Phase Analysis. IEEE Trans. Inf. Forensics Secur..

[11] Nicolalde-Rodríguez D.P., Apolinário J.A., Biscainho L.W. Audio authenticity based on the discontinuity of ENF higher harmonics. Proceedings of the 21st European Signal Processing Conference.

[12] Esquef P.A.A., Apolinário J.A., Biscainho L.W. (2014). Edit detection in speech recordings via instantaneous electric network frequency variations. IEEE Trans. Inf. Forensics Secur..

[13] Esquef P.A.A., Apolinário J.A., Biscainho L.W.P. Improved edit detection in speech via ENF patterns. Proceedings of the IEEE International Workshop on Information Forensics and Security (WIFS).

[14] Min-Dianey K., Le T., Choi J., Pham P. (2021). Advanced Optical Detection through the Use of a Deformably Transferred Nanofilm. Nanomaterials.

[15] Min-Dianey K., Le T., Qadir A., M’Bouana N., Malik M., Kim S., Choi J., Pham P. (2021). The Ripple Effect of Graphite Nanofilm on Stretchable Polydimethylsiloxane for Optical Sensing. Nanomaterials.

[16] Lin X., Kang X. Supervised audio tampering detection using an autoregressive model. Proceedings of the IEEE International Conference on Acoustics, Speech and Signal Processing (ICASSP).

[17] Fechner N., Kirchner M. The Humming Hum: Background Noise as a Carrier of ENF Artifacts in Mobile Device Audio Recordings. Proceedings of the Eighth International Conference on IT Security Incident Management & IT Forensics.

[18] Hajj-Ahmad A., Wong C.W., Gambino S., Zhu Q., Yu M., Wu M. (2019). Factors Affecting ENF Capture in Audio. IEEE Trans. Inf. Forensics Secur..

[19] Brixen E.B. Further investigation into the ENF criterion for forensic authentication. Proceedings of the 123rd Audio Engineering Society Convention.

[20] Garg R., Varna A., Wu M. ’Seeing’ ENF: Natural time stamp for digital video via optical sensing and signal processing. Proceedings of the 19th International Conference on Multimedea.

[21] Selesnick I.W., Odegard J.E., Burrus C.S. Nearly symmetric orthogonal wavelets with non-integer dc group delay. Proceedings of the IEEE Digital Signal Processing Workshop Proceedings.

[22] Haykin S.S. (2002). Adaptive Filter Theory.

[23] Box G.E., Jenkins G.M., Reinsel G.C., Ljung G.M. (2015). Time Series Analysis: Forecasting and Control.

[24] Chang C.C., Lin C.J. (2011). LIBSVM: A library for support vector machines. ACM Trans. Intell. Syst. Technol. (TIST).

[25] Quiros A.R.F., Bedruz R.A., Uy A.C., Abad A., Bandala A., Dadios E.P., Fernando A. A kNN-based approach for the machine vision of character recognition of license plate numbers. Proceedings of the Tencon IEEE Region 10 Conference.

[26] Hinton G.E., Osindero S., Teh Y.W. (2006). A fast learning algorithm for deep belief nets. Neural Comput..

